# Why are bacteria different from eukaryotes?

**DOI:** 10.1186/1741-7007-11-119

**Published:** 2013-12-13

**Authors:** Julie A Theriot

**Affiliations:** 1 Department of Biochemistry, Stanford University School of Medicine, Stanford, CA 94305, USA; 2 Department of Microbiology and Imxxmunology, Howard Hughes Medical Institute, Stanford University School of Medicine, Stanford, CA 94305, USA

## 

**Figure:**
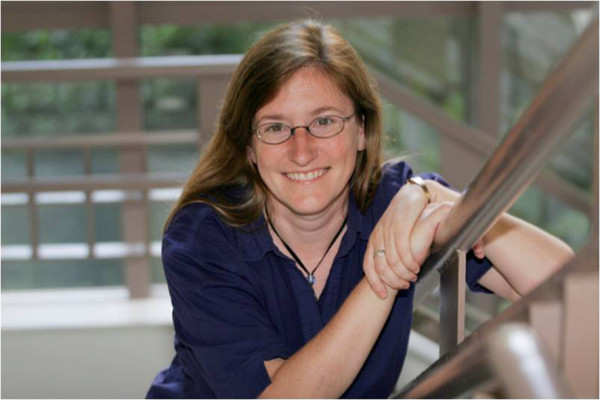
**Julie Theriot**.

## 

Julie Theriot graduated from the Massachusetts Institute of Technology as a double major in biology and physics, and her career as a biologist ever since has been notable for the quantitative rigor of her approach to the messy world of biology. As a graduate student at the University of California San Francisco, she began studying the subversion of actin polymerization by pathogenic bacteria in animal cells, and more general issues of bacterial and eukaryotic motility remain the focus of her group’s research at Stanford University. With colleagues Rob Phillips, Jane Kondev, and Hernan Garcia, she has published a textbook, Physical Biology of the Cell, exploring the applications of mathematical and physical modeling in cell biology. In the interview here, she applies a breathtaking breadth of scholarship and a fearless imagination to the fundamental question of the difference between bacterial cells and ours.

## Aren’t more and more similarities being found between bacterial cells and eukaryotic ones? How different are they in fact?

It is true that over the past 15 or 20 years we have identified a surprisingly large number of molecular similarities between bacterial cells and eukaryotic cells. Of course we have known about the profound similarities across the entire phylogenetic tree of life in many of the machines of the central dogma (ribosomes, polymerases, and so on) and the enzymes of central metabolism, but now we’ve also found homologs of the major eukaryotic cytoskeletal proteins in bacteria and many other surprises. But it is still a fundamental observable fact that the vast majority of bacterial cells are physically small and morphologically simple compared with the vast majority of eukaryotic cells. There are certainly exceptions to this - there are bacteria that are large and complicated and there are eukaryotes that are small and simple - but if you just look at any random bacterium versus a random eukaryote, it is clear that there is a fundamental quantitative and qualitative difference in size and complexity. Archaea, which make up the third major domain of life, have some molecular signatures that seem quite similar to those in eukaryotes [1], but morphologically they look very much like bacteria. Indeed this is the reason that we didn’t recognize them as a distinct domain until very recently [2]. The overall argument about the origins of morphological complexity that I want to make here applies equally to bacteria and archaea, but I’m going to focus on bacteria for specific examples just because we know so much more about them.

The most obvious difference between eukaryotes and bacteria is that there is a membrane-bounded nucleus in eukaryotes and not in bacteria - again, for the most part: there is a bacterium with the wonderful name *Gemmata obscuriglobus* that is described as having a double membrane enclosing the DNA in a nucleus-like structure [3], although the structure is apparently contiguous with the plasma membrane [4], so in that sense it is very different from a eukaryotic nuclear membrane and this is certainly a special case. But leaving that example aside, the main consequence biologically of having a membrane-enclosed nucleus is that transcription and translation are uncoupled. So there is a fundamental kinetic and organizational difference between eukaryotes and bacteria in the way that genetic information is expressed in the form of protein and is therefore allowed to be converted into cellular structure, function and organization.

## So how does that affect the function of bacterial and eukaryotic cells?

Well, let’s now think a little bit about what other cellular features go along with a membrane-enclosed nucleus. Another major difference between eukaryotes and bacteria is the proliferation of other membrane-bounded organelles, of which you see many different kinds within single eukaryotic cells - for example, the Golgi apparatus, the endoplasmic reticulum, and so on. Again, there are a few bacteria that have internal membranes, although in most cases those membrane-enclosed organelles in bacteria are contiguous with the plasma membrane, like the pseudo-nuclear membrane of *Gemmata*. One example is the magnetosomes of the bacterium *Magnetospirillum magneticum*; these are little crystals of magnetite wrapped inside of membrane invaginations that the cells use to orient themselves along the earth’s magnetic field lines [5]. Because these structures are continguous with the plasma membrane, they don’t really act as topologicaly separate compartments. A critically important exception is the cyanobacteria, which carry out photosynthesis in the elaborate thylakoid endomembrane system. The thylakoids do appear to be truly separate from the plasma membrane and can be topologically quite complicated [6]. But although we know quite a lot about the mechanisms of photosynthesis in the thylakoids, we know relatively little about membrane traffic in these organisms, so I can’t really comment on how similar their organizational mechanisms are to eukaryotic endomembranes. Going along with the proliferation of membrane-enclosed organelles in eukaryotes is usually a higher degree of subcellular compartmentalization, of assigning different kinds of functions to different regions of the cell. And of course, eukaryotes have endosymbionts, the mitochondria and chloroplasts that used to be bacteria that the eukaryotes have taken into themselves and tamed for their own purposes [7].

Another major observable difference is that eukaryotic cells are able to make very big, fancy, multicellular organisms like redwood trees and elephants. Among the three major groups of macro-organisms (those visible to the naked eye), animals and plants are the better studied, but the largest fungi are also remarkable for their vast size and lifespan [8]. Bacteria can also form multicellular structures, such as biofilms, that require complex intercellular signaling and developmental programs, as well as deposition of extracellular matrix [9], but they do not approach the structural complexity of eukaryotic multicellular organisms. The largest of the bacterial communities are formed by cyanobacteria and are called stromatolites; these are made up of beautiful layered structures that form through cycles of bacterial growth, matrix deposition, and accretion of mineral particles [10,11]. Stromatolite structures, though, have remained fundamentally unchanged for over three billion years, as stromatolites make up the oldest recognizable fossils of living organisms. They flourished until the Cambrian explosion, when they became much more rare as, presumably, the newly evolved animals began to crawl around and nibble on them. In support of this idea, stromatolites became more abundant in the fossil record after the major extinction events that wiped out most of the animals, and then receded again when the animals bounced back [12]. Today the only living stromatolites are found in extremely salty bays that are hostile to animal life. So I would say qualitatively in terms of complexity as well as direct competition, true and highly evolvable (and apparently hungry) multicellularity is a feature of the eukaryotes, not of the bacteria.

Finally, and I think not coincidentally, eukaryotes typically have genomes that are greatly expanded in length by as much as several orders of magnitude beyond those of bacteria, and those genomes usually contain a lot more noncoding DNA whose function we don’t understand.

Can you explain why eukaryotes have such an expanded genome, given that we don’t think most of it is doing much or we don’t know what it’s doing?

Sadly I don’t have an answer to that question, and as you know the possible function of noncoding DNA is an intensely controversial area right now [13,14]. I will point out that it has been known for quite a while that genome size in a wide variety of organisms seems to correlate better with cell size than with number of protein-coding genes or apparent complexity [15], so if cell size itself is a selectable trait that might be part of the answer. But what I am going to try to explain is why eukaryotes do not seem to worry about how much extra DNA they are carrying around. In principle that opens an opportunity for picking up more genes and more chromosomes, more bits of DNA whose function may not yet be obvious to us, but may well be important to the cells that are carrying it.

## Or might evolve

Yes, or might evolve. Having the capacity to carry around and segregate lots and lots of DNA also just gives the eukaryotic cells more options and more flexibility.

The much larger cell size for eukaryotic cells, which seems to be connected with all of the other differences between eukaryotes and bacteria, brings up the issue of the diffusion limit, which Kevin Young wrote about in his contribution to the Forum you recently published on cell size [16]. That was a terrific article, and I agree with everything he said, but I think he didn’t take the argument quite far enough, and that’s what I’m going to do next. His essential point was that bacterial size and structure are constrained by the need to import nutrients efficiently and divide accurately through mechanisms that depend only on diffusion. Even some of the largest bacterial cells we know are still effectively diffusion-limited; for example, *Thiomargarita namibiensis* appears as a sphere up to 750 μm across, easily visible to the naked eye, but is organized as a very thin shell of cytoplasm, less than 2 μm thick, surrounding a gigantic vacuole [17]. But as soon as you can set up an intracellular molecular transport machinery such as a filamentous cytoskeleton and associated molecular motors, then having the genome be readily accessible to diffusive transport becomes less of an issue, freeing up eukaroytic cells to become physically large.

What we’d really like is some simple, cogent explanation that ties all of these eukaryotic features together: the membrane-enclosed nucleus, the elaboration of other topologically separate membrane-bound compartments, the ability to capture endosymbionts, the ability to make fancy multicellular organisms, the greatly expanded genome, and the large cell size. When I was in graduate school, the explanation was known and it was very straightforward. It was that eukaryotes have a cytoskeleton and bacteria do not. If you go down the list of all the things that are special about eukaryotic cells, you can ascribe virtually all of them to functions of the cytoskeleton. For example, you need structural elements, including microtubules, to organize the membrane-enclosed nucleus and the extensive internal membrane system. And coming back to the expanded genome, we can see that it is simple to divide if you have a mitotic spindle, because adding another chromosome, or even doubling or quadrupling the size of your genome, is no big deal; the mitotic spindle can take care of segregating extra chromosomes using the same mechanism that it uses to segregate just a few. This is because eukaryotic spindles use essentially the same microtubule-kinetochore interface structure repeated for every chromosome, and the collective decisions such as when to enter anaphase are carried out by checkpoint machineries that enforce the rule that all of the kinetochores must be attached before the next step can proceed [18]. In contrast, bacteria that have multiple chromosomes seem to segregate them by using independent, orthogonal machineries specific for each chromosome [19], and don’t appear to have anything as general or as scalable as a mitotic spindle.

Turning to the actin cytoskeleton, this is also vital for many of the eukaryotic-specific features we have discussed. Dynamic actin assembly and disassembly are necessary for phagocytosis, to separate a large membraneous organelle from the plasma membrane compartment, and to also capture an endosymbiont [20]. And then to make a multicellular organism, you need two kinds of interactions between cells. First, you need the ability to lay down an extracellular matrix, which bacteria are also perfectly capable of doing. But then you need some kind of structural elements within cells that can connect to the extracellular matrix and to one another in such a way that forces can be continuously transmitted from the cells to the matrix and from one cell to another. This is the property that is necessary for cells to make simple tissues such as epithelia, where sheets and ensembles of cells can get bigger and bigger and perform coherent behaviors. In animal cells, these processes rely on the actin cytoskeleton [21], and there is evidence that similar cytoskeleton-based processes are also necessary for simpler kinds of multicellularity in non-metazoan eukaryotes such as *Dictyostelium*[22] and *Volvox*[23].

The problem with this argument about the basis of the difference between eukaryotes and bacteria is that it all depends on bacteria not having a cytoskeleton, which is what we believed in the early 1990s. But then it was discovered by several very convincing converging lines of evidence, spearheaded by Joe Lutkenhaus, that the bacterial protein FtsZ, which forms a ring around the middle of the bacterial cell and has an essential role in cell division [24], is a homolog of tubulin [25,26]. And when the atomic structures for both tubulin and FtsZ were solved at the same time, it was absolutely clear that they were nearly superimposable and almost certainly true homologs in the sense of being derived from a common ancestor [27,28]. So there went the assumption that bacteria do not have a cytoskeleton.

My research up until that point had focused on the actin cytoskeleton, so for a little while I could maintain my eukaryotic-centric world view by saying to myself that bacteria have tubulin but they don’t have actin, and so that must be the most important difference between us and them. But then a few years later, in a series of quite spectacular papers where the cell biological evidence for the shape-determining role of a certain class of bacterial actin-like proteins including MreB [29], was staggeringly confirmed by the undeniable structural similarity between MreB and actin [30], it was quite clearly demonstrated that bacteria do in fact have actin homologs. In the 10 years or so since that discovery, a lot of people have been searching for more different examples of actin and tubulin homologs in bacteria, and indeed we can find a tremendous number of such homologs, a vast proliferation with different biological functions, with various actin homologs like ParM involved in plasmid segregation [31] and MamK necessary for magnetosome alignment [5]. I’m particularly fond of the work of Joe Pogliano, who has gone searching for actins and tubulins carried by plasmids and bacteriophages, and has found an outrageously big zoo of both actins and tubulins [32,33]. And in a few bacteria, there is even some evidence that they have homologs (or at least functional analogs) of intermediate filament proteins [34]. So we must absolutely acknowledge that the major eukaryotic cytoskeletal proteins are also present in our bacterial comrades, indeed there are many copies of them with distinct biological functions.

So I would like to rephrase the question about what the difference is between eukaryotes and bacteria. We now know that everyone has a cytoskeleton, but still there are fundamental and easily observable morphological differences between these two domains of life, where eukaryotes have used their cytoskeletons to get larger and more morphologically complex and even truly multicellular, while bacteria basically have not done so. So the question I’d really like to ask is, if bacteria have a cytoskeleton, why don’t they do anything more interesting with it?

## And are you going to explain why bacteria don’t do what we do with our cytoskeletons?

I am. At least, I have a hypothesis. It is an untested hypothesis, but I’ve been thinking about this now for a few years, and there is a lot of supporting evidence. I think it is at least a unifying concept that I hope will be provocative, and perhaps lead to experiments and analysis that might really test this idea.

The starting point for my hypothesis is that the central feature of the cytoskeletal elements that are universally shared among organisms, and are necessary for cellular life, is the ability to form protein polymers that can give rise to large-scale cell organization and cell division via the dynamic assembly and disassembly of helical protein filaments. That is found everywhere. Besides the actin- and tubulin-related cytoskeletal proteins in bacteria, there are structures like bacterial flagella and bacterial pili, which are also fundamentally helical homopolymers of proteins. Bacteria are perfectly good at making those kinds of structures. They are perfectly good at governing the dynamics of those structures. So why don’t they do anything more interesting with them? Here is my hypothesis: eukaryotes enhance the intrinsic assembly features of the helical filament protein systems with two particular kinds of cytoskeleton-associated factors, which have not yet been found in bacteria. And those two are regulated nucleators - centrioles for example - and linear stepping molecular motor proteins - the eukaryotic myosin and kinesin molecules.

**Figure 1:**
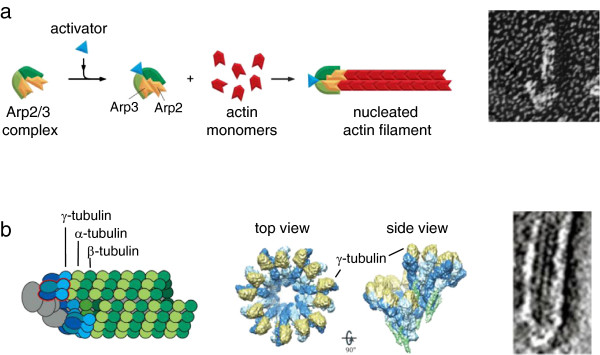
**Cytoskeletal filament nucleation by modified subunits. (a)** Nucleation of actin filaments by the Arp2/3 complex. Left: diagram of Arp2/3 complex before and after activation, showing rearrangement of actin-like subunits leading to templated filament growth (Copyright 2008 from *Molecular Biology of the Cell,* 5th edition by Alberts *et al*. Reproduced by permission of Garland Science/Taylor & Francis LLC [41]). Right: electron micrograph showing the appearance of an actin filament nucleated by Arp2/3 (at the bottom) (from *Proc Natl Acad Sci U S A*[35]). **(b)** Nucleation of microtubules by the γ-tubulin ring complex. Left: diagram of microtubule templated from a ring complex (Copyright 2008 from *Molecular Biology of the Cell,* 5th edition by Alberts *et al*. Reproduced by permission of Garland Science/Taylor & Francis LLC [41]). Middle, structure of the ring complex by cryo-electron microscopy, showing how the γ-tubulins are held in the proper configuration to imitate a microtubule plus end (reprinted by permission from Macmillan Publishers Ltd: *Nat Rev Mol Cell Biol***12:**709**–**721, copyright 2011 [38]). Right, electron micrograph of the end of a microtubule nucleated from a ring complex (reprinted by permission from Macmillan Publishers Ltd: *Nat Cell Biol***2:**365**–**370, copyright 2000 [42]).

For actin, the best-characterized of the regulated nucleators is the Arp2/3 complex, which has two actin-related proteins as part of the complex and then five other proteins that hold them together [35] (Figure 1a). In its isolated form, the two actin-related proteins of the Arp2/3 point off in slightly different directions [36], but when the complex is activated for its nucleation activity they swing around to imitate the starting point of the two protofilaments of the actin filament structure, and this structural mimicry of the growing tip of an actin filament is probably the basis of the nucleating activity for the Arp2/3 complex [37]. For microtubules, the best characterized nucleator is the γ-tubulin ring complex, which has 13 copies of the protein γ-tubulin (a paralog of α- and β-tubulin) and then some other proteins that hold them in a slightly distorted ring that can template the growth of a microtubule with 13 protofilaments [38,39] (Figure 1b). There are other actin nucleators and there are other microtubule nucleators that operate by different mechanisms. But it seems from those two examples that a very reasonable way to regulate the initiation and assembly of helical cytoskeletal polymers is to just make another copy of the gene for the subunit and then allow it to specialize a little bit so that it becomes a regulatable nucleator. Certainly that is the sort of thing that bacteria could do if they wanted. They would have no problem duplicating and modifying the genes for the cytoskeletal proteins, as they have demonstrated with the proliferation of the different flavors of actin and tubulin homologs that are used in such a wide variety of contexts. For example, *Bacillus subtilis* has three different chromosomally encoded paralogs, each of which is homologous to actin, MreB, Mbl, and MreBH, that appear to have somewhat overlapping functions [40]. But so far, we do not know of any specialized actin- or tubulin-related proteins in bacteria that are used specifically as regulated nucleators for their main self-assembling subunits MreB and FtsZ.

## So why don’t bacteria want regulated nucleation?

This is the corollary to my argument. If my hypothesis that bacteria do not have regulated cytoskeletal nucleation proteins is true - and I will go through the cell biological evidence that makes me think this is true - then the question is whether they really do not want to have them or whether they just never had the opportunity to develop them. I think, at least as far as nucleators go, the opportunity to develop them is not a very high barrier. So I think it must be that bacteria simply have a fundamentally different strategy for cytoplasmic organization as compared to eukaryotes.

## What makes you say it’s not a high barrier? Do we have evidence that it’s happened more than once in eukaryotes?

I don’t have good evidence that forming nucleating factors by duplication of the subunits has happened more than once for each of the two major cytoskeletal structures because both the Arp2/3 complex [43] and the γ-tubulin ring complex [44] are very well conserved across all eukaryotes, so it is most likely that the relevant duplications happened fairly early in the eukaryotic lineage and have been maintained ever since. However, at least in the case of actin, there are many different, distinct molecular families of nucleators that can operate by different but equally simple mechanisms. For example, the actin nucleators Spire [45] and Cordon-bleu [46] both appear to nucleate actin by having a series of three or four domains that bind directly or indirectly to actin monomers; these domains can bring the actin subunits into close enough proximity and appropriate enough orientation to get over the kinetic barrier to actin nucleation and start the growth of a filament. In the particular case of this category of nucleators, I am quite confident that bacteria would be able to develop them if they wanted to, as indeed two bacterial pathogens are known to express secreted virulence factors that act as host cell actin nucleating factors by exactly this mechanism [47,48]. For these virulence factors, it is not clear whether the pathogens picked up their actin nucleators by horizontal gene transfer or by convergent evolution, but in either case it is still striking that bacteria are easily able to nucleate eukaryotic actin filaments but do not seem to have any regulated protein nucleators for their own cytoskeletal filaments.

But the thing that I think is really interesting about cytoskeletal filament nucleation in this context is that classically when we were taught the theory of protein polymerization from Fumio Oosawa [49,50] and Terrell Hill [51,52] and all those giants in the field, their argument was that it is important, kinetically, that nucleation be the rate-limiting step for polymer formation. And that is indeed observably true for actin and for microtubules and for the bacterial flagellum, the classical examples of helical protein self-assembly that they were trying to describe with their comprehensive theoretical treatments. But when people started doing very careful kinetic studies on the bacterial cytoskeletal proteins - and this I think has been done best for FtsZ [53] and for ParM [54] - it became clear that nucleation for the bacterial cytoskeletal proteins is actually very, very fast. It’s spontaneous. The way bacterial cells regulate where they have their filaments is not by regulating the site of nucleation, but rather by regulating the sites of stabilization and destabilization of spontaneously nucleating filaments.

For those of us who have been raised on the thermodynamic theory of protein polymerization in the context of cell biology, this is deeply shocking. Spatial localization of cytoskeletal components in bacteria simply appears to use a fundamentally different mode of organization from the one we see for all of the organized cytoskeletal assemblies in eukaryotes, and frankly we as cell biologists are justified in being a little bit freaked out.

## These are mechanisms that regulate fundamental processes, aren’t they? This is bacterial cell division?

Yes, that’s right. The dynamic cytoskeletal polymers found in bacteria seem to be just as important to the bacterial cells as they are to us eukaryotes, and they are involved in similarly crucial cell biological processes. Also the bacterial cytoskeletal proteins are very widely distributed among bacteria and even archaea [55,56]. So again, my premise is that since we must now accept that bacteria do have a dynamic cytoskeleton, we must now try to understand why they don’t do something more interesting with it, and when I say 'interesting’ I mean in my eukaryotic-centric view becoming larger, more morphologically complex, or multicellular. I absolutely do not mean to disparage the many very interesting things that bacteria do and have done in their evolutionary history. The cyanobacteria invented oxygenic photosynthesis for which I am very grateful, and in general bacteria have much more interesting twists on metabolism than do us chemically unimaginative eukaryotes. But I do realistically claim organismal size, morphological complexity, and true multicellularity as eukaryote-specific features that deserve explaining.

**Figure 2:**
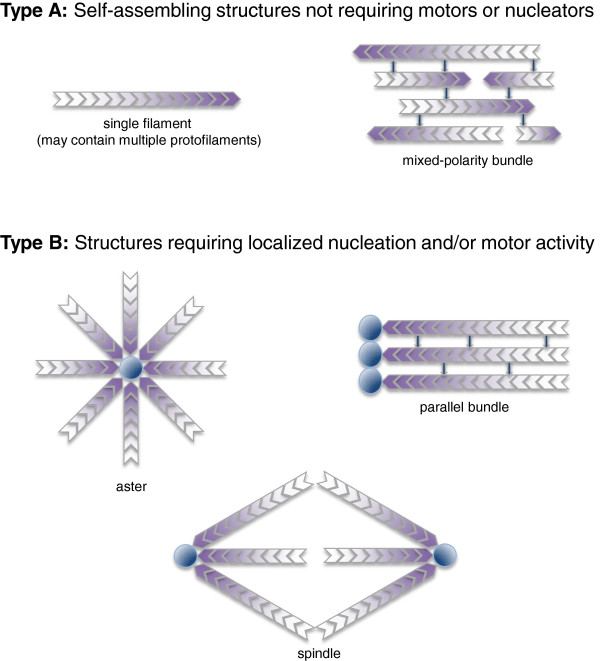
**Types of cytoskeletal filament arrays.** Type **A**: simple filament arrays that can self-assemble in the absence of spatially regulated nucleators or molecular motor protiens. Shading indicates the orientation of filament polarity. Type **B**: complex filament arrays that require either nucleation or motor protein activity, or both. Dark circles represent nucleators.

As we delve into the details of my argument I will delineate a few of the many biological examples of well-understood systems that have convinced me that bacteria simply do not have cytoskeletal nucleators or cytoskeletal motor proteins as we understand them in eukaryotes. At present, I hope you’ll bear with this assertion for just a bit, so that I can more fully explain my hypothesis. If you’ll accept for the moment my premise that the real difference between bacterial cells and eukaryotic cells lies in the eukaryotic proliferation of cytoskeletal nucleators and molecular motor proteins, then a relevant question becomes, what kinds of cellular structures can you make if you have nucleators and motors versus the structures that you can make if you don’t? The diagram in Figure 2 shows - given some reasonable assumptions about the universality and fundamental nature of helical protein filament assembly - what larger-scale structures you can get with and without nucleators and motors. In particular these drawings show structures that can be formed by polarized cytoskeletal filaments, where the subunits assemble in a head-to-tail fashion so that the two ends of the filaments are structurally distinct. According to the basic theories of protein polymerization, this is expected to give a polymer where the kinetics of subunit addition and loss at the two ends are also distinct, where one end grows and shrinks more quickly than the other [51]. In microtubules, the fast-growing end is called the plus end and the slow-growing end is called the minus end. In actin filaments, the fast-growing end is called the barbed end and the slow-growing end is called the pointed end. (Incidentally, both the Arp2/3 complex and the γ-tubulin ring complex nucleate their cognate filaments from the slow-growing end.)

The simple structures that can be made from polarized filaments I will call type A structures. In the absence of nucleators you can obviously make a single filament of essentially any length and that single filament can have many protofilaments. A microtubule is a single filament with 13 protofilaments that can be arbitrarily long. A bacterial flagellum is also a single filament that happens to have 11 protofilaments, and flagella can also be very long - 10 microns long *in vivo*. Both of these structures self-assemble quite nicely from solutions of purified protein monomers; indeed these were the examples that have formed much of the basis of our understanding of the fundamental thermodynamics of protein polymerization [50]. So those kinds of structures you can make regardless of whether you are a bacterium or a eukaryote and regardless of the presence of nucleators or motors. The other kind of structure that is very easy to make is a mixed polarity bundle. In crowded solutions, such as in the cytoplasm of a living cell, colloidal rods will tend to align with one another simply because of entropy and excluded volume effects [57]. When the rods happen to be cytoskeletal filaments, they can easily form bundles either by interacting with one another laterally, or else by having cross-linking proteins that help pull them together. For the bacterial cytoskeleton, the clearest example of a mixed polarity bundle is the plasmid-segregating actin homolog ParM, which can assemble into mixed polarity bundles on its own [58]. It is also very likely that the FtsZ ring in bacterial cytokinesis is essentially a mixed polarity bundle, formed with the help of cross-linking proteins [59].

The kinds of structures for which I think, theoretically, you need to have either localized nucleation or motor activity, or both, the type B structures, are structures like asters, where many cytoskeletal filaments with the same polarity emanate from a single location, or parallel bundles of filaments, where all of the filaments are pointing in the same direction. If filaments form spontaneously and then come together through purely entropic effects, there is no intrinsic reason for them to assemble in a particular orientation. So if you want to have a parallel bundle, such as in a muscle sarcomere, you have to control the assembly or orientation of the filaments, for example by having them all nucleated from the same site. And of course a great example of all of these properties is the mitotic spindle, where you have parallel bundling and anti-parallel bundling of microtubules, and also their nucleation from particular sites at the spindle poles.

My assertion, and I’ve really scoured the literature here, is that no type B structures - asters and parallel bundles and spindles - have been observed in the cytoplasm of bacteria (with one very interesting exception which is I think the exception that proves the rule - and I’ll come back to that a bit later). There are plenty of examples of single polarized filaments in bacteria. There are plenty of examples of mixed polarity filament bundles in bacteria. But the type B structures are critical I think to making eukaryotes what we are today, by allowing the elaboration of the microtubule cytoskeleton to give complex organelle dynamics and fabulously flexible DNA segregation capacity, and elaboration of the actin cytoskeleton to give us the possibility of amoeboid motion and phagocytosis, which allow us to run around and eat all those pesky bacterial biofilms and tame endosymbionts. And then once we have those kinds of structures and mechanisms, we are able to overcome the diffusion barrier and the increase in size and complexity of eukaryotic cells follows naturally from that.

That’s the hypothesis. The supporting details can be discussed from three different perspectives. The first focuses on self-assembly dynamics, and the rules about the kinetics and thermodynamics of self-assembly that come from the intrinsic properties of proteins - can these really be different between bacteria and eukaryotes? The second perspective focuses on the nucleators - is it true that bacteria don’t have them? And if not, why not? And then the third perspective is all about the motors - is it true that bacteria don’t have them? And if not, why not? And beyond that, there are also other possible explanations besides the cytoskeletal hypothesis for why eukaryotes and bacteria are different; this is a fourth level, even more general and more speculative, but one that I think helps tie this whole story together.

## I think it would be good to know all four supporting arguments for your hypothesis. Can we start with number one?

The first thing to think about is the question of protein self-assembly, because classically, when we think about the cytoskeleton, we imagine lots of little subunits that are able to assemble in an oriented fashion, to make larger structures. The ability of proteins to form homo-oligomers is very prevalent and, in fact, I would say it is almost the default thing for proteins to be able to do. There have been some genome-wide studies showing, for example, that in *Escherichia coli*, if you look at the known protein oligomers (and of course there may be some we don’t know), something like 80% of them are homo-oligomers, where proteins assemble with other copies of themselves [60]. Structural biologists have done a very nice job of breaking down the kinds of symmetries you can get in these homo-oligomers into different kinds of classifications. Really making a helix is just one particular phylogenetic group, if you will, of the kinds of structures that proteins can make by self-assembly.

**Figure 3:**
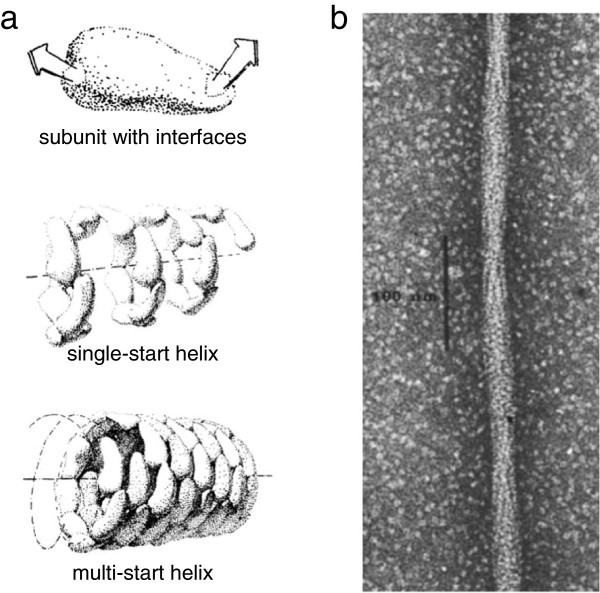
**Helical protein filaments formed by self-assembly. (a)** General scheme for protein self-assembly into helices. For any globular protein of arbitrary shape, as shown at the top, considered as interacting with a second copy of itself in all possible orientations, there will be some pair of surface patches that result in optimal binding energy. It is highly unlikely that those two interface patches will happen to reflect any specific geometrical symmetry. When many copies of the same subunit self-associate by binding to one another through these surface interactions, a one-start helix with a single protofilament is the default structure formed, as shown in the middle. At bottom, if weaker interactions can also form laterally between subunits, multi-start helices may be stabilized (adapted with permission from the Royal Society of Chemistry [62]). **(b)** Electron micrograph showing a single filament of sickle-cell hemoglobin (HbS) (reprinted by permission from Macmillan Publishers Ltd: *Nature***272:**506**–**510, copyright 1978 [63]).

Now there are two really nice things about helices. One is that a helix enables you to make structures of variable length, while most other oligomer types make a closed structure with a defined size, such as a viral capsid. But a helix that grows by addition of subunits onto the end can in principle be tuned over a very wide size (or length) range. The second thing that’s nice about the helix as a mode for protein self-assembly was pointed out originally by HR Crane in 1950 [61] and then followed up by Linus Pauling in 1953 [62]. They used protein structural arguments to explain that when you allow many copies of the same protein to aggregate together you can hardly help but make a helix (Figure 3a). If you allow a protein to self-assemble, a helix of some kind is going to be the default. It is actually going to take more effort, in an evolutionary sense, to try and make something that’s not a helix.

## And in fact, mutant hemoglobin makes helical fibers, doesn’t it?

Yes, hemoglobin is a terrific example. Hemoglobin, of course, has been selected through evolution to be extremely soluble, so that within a red blood cell you can have 300 mg/ml of this one protein, which is an outrageously high concentration. In sickle-cell disease, a single point mutation in hemoglobin changes one charged residue on the surface to a neutral residue [64], and now in this dense cellular bag of the erythrocyte, filled almost entirely with one protein, you have a condition where the oxygen-depleted form of hemoglobin is able to self-assemble into a spectacularly beautiful helical structure with 14 protofilaments that looks absolutely classically like a microtubule or some other cytoskeletal filament [63] (Figure 3b). Sickle-cell hemoglobin is, of course, a very famous example of many principles of protein structure and function, but in this particular case it clearly shows that when you take a very soluble protein and create a condition in which it is not quite soluble, a helix is what you get. That’s the default.

If any old protein will assemble into a helix, then what is special about the cytoskeletal proteins? There are several possible answers, but one that I find compelling is that the common feature of the universally conserved cytoskeletal proteins - the actin superfamily, the tubulin superfamily - is that both of them are nucleotide hydrolases. They use the energy of nucleotide hydrolysis to switch between at least two distinct conformations. One of those conformations has a lower energy barrier to forming a filament than the other one. What this means is that if you can couple nucleotide hydrolysis kinetics to the interactions that the protein can form when it is in a helix, you can use the energy of nucleotide hydrolysis to regulate stability [65]. You can have the filaments assemble when the subunits have the ATP or GTP bound, and then after hydrolysis takes place, the energy released by hydrolysis is stored in the lattice in such a way that now disassembly becomes favorable. And this means that within a cytoplasm, where you have a good supply of ATP and GTP, you could have constantly dynamic filaments without having to change the concentration of anything.

## So are you going to suggest that bacteria don’t have the energy to regulate filament assembly?

**Figure 4:**
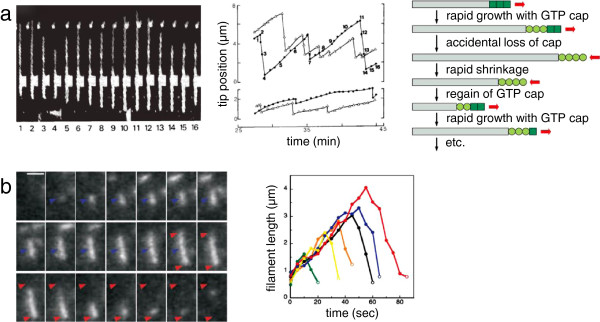
**Dynamic instability of cytoskeletal filaments from eukaryotes and bacteria. (a)** Dynamic instability of eukaryotic microtubules. Left: direct observation using dark-field microscopy of a microtubule undergoing dynamic instability. Middle: graph showing position of plus ends (top) and minus ends (bottom) for two dynamically unstable microtubules, with repeated cycles of growth and shrinkage. Numbered points correspond to individual video frames as labeled on the left (reprinted by permission from Macmillan Publishers Ltd: *Nature***321:**605**–**607, copyright 1986 [69]). Right, schematic diagram showing the connection between nucleotide hydrolysis and dynamic instability (Copyright 2008 from *Molecular Biology of the Cell,* 5th edition by Alberts *et al*. Reproduced by permission of Garland Science/Taylor & Francis LLC [41]). **(b)** Dynamic instability of bacterial ParM filaments. Left: fluorescence time-lapse images of a single ParM filament over time. Blue arrowhead shows position of initial filament appearance; red arrowheads mark the most extreme positions of the two tips. Video frames are separated in time by 5 s; scale bar is 2 μm. Right, traces of filament length over time for six different ParM filaments, showing a phase of growth followed by catastrophic shrinking. (From Garner EC, Campbell CS, Mullins RD: Dynamic instability in a DNA-segregating prokaryotic actin homolog. *Science* 2004, **306:**1021**–**1025. Reprinted with permission from AAAS [54].)

Absolutely not. Bacteria have a ton of energy; I don’t know of any cases where ATP availability is limiting for any normal biological process. And in fact bacteria use the cycle of nucleotide hydrolysis to modulate the assembly of their cytoskeletal filaments quite nicely. This is not the difference between bacteria and eukaryotes. If you look at the dynamics of, for example, FtsZ, it turns over very fast, even in the cytokinetic ring. You can see a beautiful ring that persists stably for some minutes before cytokinesis and before the cells separate [66], and yet there are very convincing photobleaching studies showing that the filaments within that ring are continuously turning over just like the microtubules in a mitotic spindle, or the actin filaments in a lamellipodium. Indeed it has been shown that mutants in FtsZ that have slowed GTP hydrolysis kinetics also have a slower turnover rate inside the living cell [67]. ParM, which is the very well characterized actin homolog that is used to segregate plasmids in bacteria [31], even shows dynamic instability [54], which is one of the classic outcomes of the coupling of assembly to nucleotide hydrolysis for eukaryotic cytoskeletal filaments [65,68-70]. I think it is very clear that those intrinsic, dynamic properties of the self-assembling filaments - the coupling to nucleotide hydrolysis, the rapid turnover, kinetic properties like dynamic instability - those things are universal in cellular cytoskeletons (Figure 4). That is not a problem for bacteria, and that is not the difference between bacteria and eukaryotes.

Moving on to the second perspective for my argument, if helical protein self-assembly regulated by nucleotide hydrolysis is universal, then what can we say about the role of regulated nucleation of cytoskeletal filaments in determining the difference between bacterial and eukaryotic cell organizational strategies? Here I think we are digging into much richer soil. I briefly mentioned this earlier, but now I’d really like to emphasize the striking observation that both FtsZ (bacterial tubulin) and ParM (bacterial actin) nucleate like mad [53,54]. As a cell, you would really have to put a lot of effort into not nucleating them.

For ParM, the filaments undergo very rapid dynamic instability and shrink back to nothingness unless they are stabilized by encountering cognate segments of DNA bound by the correct protein partner, both of which are normally found on the plasmid that is using ParM for segregation [71]. This mechanism rather neatly ensures that ParM filaments forming in a cell will be stabilized to push the plasmids apart only when there are two copies of the plasmid present, one to stabilize each end of the normally unstable filament. For FtsZ, its major regulator is a destabilizing factor, MinC [72], which undergoes its own very fascinating form of spatial regulation, but the short version is that the FtsZ ring that initiates bacterial cell division can form only where MinC is not; that is, FtsZ nucleation is spontaneous, but filament stability is regulated.

If we had much more time to talk, I’d also tell you the whole beautiful story about the spatial regulation of MinC [73]. In *E. coli*, MinC is carried around by MinD, which arguably is yet another spontaneously nucleating self-assembled polymer that doesn’t happen to be homologous to any of the known eukaryotic cytoskeletal proteins, so it is not really part of my central story here, but I can’t stop myself from mentioning it anyway, and its kinetic regulation is highly relevant. MinD self-assembles on the bacterial membrane, and the MinD filaments are then destabilized by another protein factor, MinE. The kinetic interaction between MinD assembly and MinE destabilization results in spectacular oscillatory positioning of the MinC inhibitor inside of cells [74] and self-propagating waves when reconstituted *in vitro*[75]. In brief, this impressively dynamic and very precise system that the bacterial cell uses to choose the site of division depends on the spontaneous nucleation of one filamentous structure (MinD) that is destabilized by a regulator (MinE). The biological purpose of MinD and MinE is to regulate the localization of MinC, which acts to destabilize the spontaneously nucleating tubulin homolog FtsZ. Over and over for bacterial cytoskeletal and cytoskeletal-like elements, we are seeing spontaneous nucleation followed by spatially localized stabilization or destabilization as the general organizing principle.

Again the really surprising thing here is that, for the cases that we understand well, nucleation plays no obvious part in the spatial regulation of cytoskeletal assembly for bacteria; everything where we understand the molecular details of spatial regulation regards filament stabilization and destabilization. My examples here are the best-characterized systems that we know in bacteria. For most of the other examples of bacterial cytoskeletal filaments, too little is known about their dynamics to enable us to guess how the nucleation versus stabilization equation will play out. I think it will be very, very interesting in the next few years to see if this is really a universal, decisive difference between the eukaryotes and the bacteria, or just an intriguing feature of the first few well understood systems. As we’ve already discussed, there are several simple strategies for developing regulatable nucleators for cytoskeletal filaments, either through specialization of a copy of the gene encoding the structural subunit, or just by recruiting another protein that has multiple binding sites for the structural subunits. Honestly, I really think bacteria could do that if they wanted to. But so far we do not know of any bacterial proteins that are specifically dedicated to nucleation of bacterial cytoskeletal filaments.

## So if nucleation can evolve easily, the question, again, is why didn’t it in bacteria?

I think you could argue that once you commit to a certain kind of dynamic strategy for your cytoskeletal filaments, back in the ancient past - maybe 3 billion years ago, when the modern version of FtsZ first came into being - then it’s not worth changing it. That’s possible. But there may be something else that we’re missing, that makes the domain-based choice of cellular organizational strategy more likely to be universal. In the fourth part of this argument, the wild speculation, I’ll get to what I think that might be. But as far as the nucleators go, it’s not so much that I think that bacteria can’t have them, it’s just that there’s no positive evidence yet that they do.

**Figure 5:**
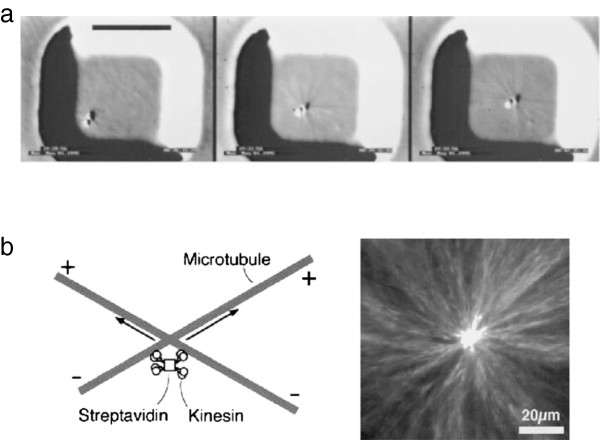
**Self-centering activity of dynamic microtubule arrays. (a)** Self-centering by nucleation and dynamic instability, for a microtubule-nucleating bead. Images are separated by 3 minutes, scale bar is 10 μm (From *Proc Natl Acad Sci U S A*[76]). **(b)** Self-centering by motors. Left: diagram of crosslinked motors reorienting microtubules. Right: fluorescence image of an aster formed in a microwell by this mechanism (reprinted by permission from Macmillan Publishers Ltd: *Nature***389:**305**–**308, copyright 1997 [79]).

There are many cases where having localized nucleators has been shown to be sufficient to give you really very interesting kinds of self-organized systems. A famous example I really like comes from experiments on dropping centrosomes or beads covered with microtubule nucleators into little microfabricated wells - you can grow up asters of microtubules and these will push the bead or the centrosome into the center of that well [76] (Figure 5a). Each growing microtubule end pushes against the wall of the well, generating a few picoNewtons of force [77], and the forces are equally balanced when the nucleating bead is near the middle. Because the microtubules are dynamic, and specifically because they are undergoing dynamic instability and occasionally shrinking back to their origin, the system does not get stuck and the centering can be maintained. This mechanism of self-centering by having centrally nucleated microtubules nudging at walls appears to be the way that the fission yeast *Schizosaccharomyces pombe* maintains the mid-cell location of its nucleus [78]. In the example of the nucleating bead in the well, we can see that just by localizing nucleation, you can set up a coordinate system that will tell you within the microchamber or within the cell where you are and which direction is inside and which is outside. If you imagine some cargo attached to a molecular motor encountering this assembly at any point in the space, the cargo attached to a minus-end directed motor such as dynein will end up in the middle, and the cargo attached to a plus-end directed motor such as kinesin-1 will go to the periphery. Going from that to being able to make something like the mitotic spindle is a relatively straightforward couple of steps, adding a second nucleating center and a protein that preferentially cross-links overlapping antiparallel microtubules, but you can’t do it at all if you don’t have the nucleator.

Now this brings me to the exception I mentioned earlier where bacterial cytoskeletal proteins can actually form a type B structure, specifically a self-centering aster. This has been seen for at least two of the eukaryotic cytoskeletal homologs associated with independent DNA elements in bacteria, an actin homolog that is encoded by a plasmid [80] and a tubulin homolog that is encoded by a bacteriophage [81]. In both cases, it appears that the self-centering activity of the associated cytoskeletal filament structures is useful to promote replication or segregation of the associated DNA element. In these cases, the plasmid or bacteriophage DNA itself is acting as the nucleating center. Other filament-forming proteins encoded by plasmids in bacteria, such as ParA, appear to help regulate the positioning of their plasmids in much the same way, even though these are not obviously homologous to one of the eukaryotic cytoskeletal proteins [82]. So it is clear that the basic mechanics for self-centering by localizing nucleation of self-assembled filaments do work just fine with the bacterial cytoskeletal and cytoskeletal-like proteins. But, and I think this is an important distinction, these structures are self-centered in more than just one way; the oriented cytoskeletal filaments do not appear to serve as tracks to provide spatial information for other cellular elements. Unlike the microtubule asters that set up a global coordinate system used by molecular motors and membrane-enclosed organelles to generate large-scale organization in eukaryotes, the plasmid and bacteriophage systems seem to operate with every man for himself. That is, they spatially localize only the very DNA element that encodes them.

This observation points out a really interesting and probably important difference between bacteria and eukaryotes that I think is fundamental. When people first started discovering all of these tubulin and actin homologs in bacteria, many of us were initially amazed at how many there seem to be, with each one apparently tuned for a single specific purpose. But maybe what we should really be amazed about is how few tubulins and actins seem to be present in eukaryotic cells. For the major filament-forming cytoskeletal subunits in eukaryotes, there may be multiple genes encoding them in any given organism, but the subunits are typically able to assemble together into a single all-purpose cytoskeleton that is used for an outrageous variety of biological processes. In eukaryotes, functional variety appears to be largely carried by the large numbers of different kinds of actin-binding and tubulin-binding proteins that are present [83,84]. Frankly it is rather extraordinary that the same kind of microtubule structure can be used to make mitotic spindles and beating cilia. As far as I can tell, this kind of creative multi-purposing of cytoskeletal filaments just does not happen in bacteria, where the rule seems to be one filament for one function.

With this in mind - the idea that eukaryotes have to deal with just one kind of actin filament and just one kind of microtubule, while bacteria juggle many kinds of each along with other cytoskeletal-like filaments such as MinD and ParA - let’s move on now to discussing the molecular motor proteins. This is the second major group of cytoskeletal regulators, after the nucleating proteins, that I suspect might simply be missing in bacteria. Like regulated nucleators, cytoskeletal motor proteins can cooperate with their filaments to generate very large-scale structures. For example, clusters of motor proteins can generate very nice organized asters *in vitro*, much as the nucleating beads do, even if their associated filaments are stabilized and non-dynamic [79] (Figure 5b). The motors, because they move toward only one end of the polarized filament substrate, are essentially able to sort out a disorganized clump of mixed-polarity filaments into something nice and orderly with uniform polarity. So the cytoskeletal molecular motors, together with localized nucleators, can make the type B cytoskeletal structures that I am arguing are so important for eukaryotic cell organization.

Obviously bacteria do have some kinds of molecular motors, if we define molecular motors very generally as just being engines that convert chemical energy into mechanical energy, which I think is a fair definition.

## You mean bacterial motors such as flagella and pili and so forth?

Yes. And the bacterial flagellar motor is just spectacular. It is an extraordinarily energy-efficient and complicated and beautiful object [85]. In fact, it is so beautiful that in the United States, the anti-evolutionary creationists seized upon it as being something so fantastic that it could not possibly have evolved [86]. Happily there is actually very nice structural evidence that evolution of the flagellar rotor has indeed occurred [87]. There are other several kinds of biological motors that can convert chemical energy into mechanical energy, and it is convenient to classify all of the biological motors we know about into five classes, which are not really mutually exclusive. The rotary motors such as the flagellar rotor would be one. Linear stepper motors, like kinesin, myosin and dynein, would be another [88]. Assemby and disassembly motors - using the forces that you get from polymerization of and depolymerization of microtubules or actin - make up another class [70]. Or there can be pre-stressed springs that are built in such a way that they store mechanical energy that can be released all at once, as, for example, in the acrosomal reaction in the horseshoe crab sperm [89]. And then there are also extrusion nozzles, where a cell will squirt out very hygroscopic polysaccharide that can allow it to jet along. *Myxococcus xanthus* does that [90].

Bacteria have some examples of all of those classes of biological motors. And they have linear stepper motors that work on DNA, or work on RNA, as substrates. What they don’t have, or at least what has not yet been found, is any linear stepper motors that work on the cytoskeletal filaments. There is nothing known that does linear stepping on FtsZ. There’s nothing known that does linear stepping on MreB or ParM or any of the other actin homologs.

## Why should bacteria not have evolved linear stepper motors?

Why should it be so difficult? Let’s take a look at the eukaryotes and see where they got their motors from. Looking just at the linear stepper motors for microtubules and actin, there are three major classes [88]. There are the myosins for actin, and the kinesins and dynein for microtubules. It has been shown structurally - and this was a real surprise for me and I think for most people - that kinesin and myosin have very similar central folds around the region where they couple nucleotide hydrolysis to piston-like motion, and are almost certainly derived from a common ancestor [91,92]. Dynein is definitely the odd man out. It is a very different kind of motor, related to a completely different class of ATPases. So I hope you’ll forgive me, for purposes of my speculative argument here, if I leave dynein aside and focus just on myosin and kinesin, and where did they come from, and why don’t bacteria have them?

It has been speculated that there was some kind of motor precursor that was the common ancestor of myosin and kinesin [93]. I don’t think that we can make any reasonable argument about which kind of cytoskeletal filament it was more likely to walk on. But the heart of both of those motors is the nucleotide switch that converts hydrolysis into a large-scale protein conformational change resulting in stepping movement. Other aspects of motor function, such as the binding to the filament, are quite different among different motors, and if you look even just within the families - the myosin family, the kinesin family - the way they couple that nucleotide switch to motion is actually very wildly, dramatically different among different individuals [94]. For example, most myosins walk toward the barbed end of the polarized actin filament, but one particular subfamily, myosin VI, walks in the opposite direction toward the pointed end [95,96]. Focusing on the nucleotide switch at the heart of the motor, these cytoskeletal molecular motors are members of what is called the P-loop NTPase family. This includes lots and lots of different ATPases and GTPases that are found in all domains of life. There has been a heroic attempt made by Eugene Koonin and colleagues to classify all of these many very divergent proteins into a reasonable phylogenetic tree based on sequence and structural similarities [97]. Given that this is such a diverse protein family spanning essentially the whole history of cellular evolution, there is some uncertainty here, but one thing about their reconstructed phylogeny really leapt out at me. According to their analysis, there is a entire branch of the P-loop NTPases that is found only in eukaryotes, and not in bacteria or archaea. This branch includes not only myosin and kinesin, but also many other critical proteins that we associate with eukaryotic cellular complexity. These include the Rho GTPase superfamily, which act as master regulators for actin cytoskeletal assembly [98], the Rab GTPases that govern many aspects of membraneous organelle identity [99], the Arf GTPases that are also associated with membrane traffic [100], the Ran GTPase that governs the directionality of nuclear import and export [101], and the heterotrimeric G proteins that influence so many aspects of eukaryotic cell-to-cell signaling [102]. So, wow. This looks very much like the list of eukaryotic-specific cellular features that we started off with.

It seems historically as if a branch of the P-loop NTPase family might have arisen in eukaryotes at some point when they had presumably already been evolutionarily separated from the bacteria and the archaea, and this novel protein family gave rise not just to the myosins and kinesins, but also to many of the regulatory and signaling proteins that we most closely associate with the eukaryotic way of life. Bacteria, of course, have very good signalling proteins, such as the large family of two-component signal transduction systems involving histidine kinases and response regulators [103]. But, bacteria just don’t seem to have the GTPases that we associate with eukaryotic signaling and large-scale cellular organization, and (particularly in animals) with complicated kinds of multicellular life.

Who knows why that happened - maybe it was just good luck, maybe the innovation that led to those branches of the P-loop NTPase superfamily is something that happened in eukaryotes so that they were able to seize advantage of it and then combine it with their other properties and develop the ability to make these very large and elaborate, well organized and polarized cytoskeletal structures that would enable them to do things like build a mitotic spindle.

So you’re arguing that there might have been a couple of relatively low-probability changes that helped eukaryotic development but weren’t important enough for bacteria to be forced to evolve that way because they could survive without it?

Bacteria already had a perfectly good strategy going without these kinds of systems. Arguably in many ways the prokaryotic side of the tree, the bacteria and archaea, are much more diverse and more successful than eukaryotes - certainly there are many more of them than there are of us. They are particularly good at diversifying their metabolisms. All of the really exciting inventions in biological chemistry, I would say, have been generated in the prokaryotic branches of the tree. Photosynthesis, for example, is simply an awesome idea, and it was cyanobacteria that came up with that. Eukaryotes never could come up with that whole crazy business about using a cubic manganese cluster to strip the electrons off of water [104]. The best that eukaryotes could do was to tame the cyanobacteria and get them to come and live inside and become chloroplasts. I think the bacterial strategy is terrific, it is just different from our eukaryotic strategy. Our strategy has much more to do with morphological diversification, including getting very large both as cells and as organisms, and developing hunting strategies of various different kinds.

## Could we come back from this prokaryotic chauvinism for a moment to the crucial differences between them and us?

OK, finally I’m going to bring this whole argument back full circle and say that really the crucial difference between them and us is the membrane-enclosed nucleus. I think this is probably both a consequence and a cause in a feedback loop mechanism of the diversification of cytoplasmic cytoskeletal structures that then gave rise to larger-scale morphological diversity in eukaryotes. This fourth part of my argument is now much more speculative than even the most speculative parts of what I have said before.

Let us stipulate that it is observable that all cells are organized in some way. What is their central organizing principle? Where is the information that is used by various different components of the cell to know where they are in relationship to everyone else? Well, if you’re a bacterium and your chromosome is in the cytoplasm, the chromosome is a spectacular source of spatial information. In most bacteria there are only one or a few chromosomes. They tend to be oriented in a very reproducible way as you go from one individual to the next [105,106] and because of the coupled transcription and translation, the physical site where you have a bit of DNA is also connected to the physical site where you make the RNA and the physical site where you make the protein from that bit of information [107]. If it is important to a bacterial cell to be able to target something to a specific location, it already has all the information it could ever hope for about which location in the cytoplasm is which because it has a well-defined, oriented chromosome present there.

Now, once you wrap that beautifully organized chromosome up in a nucleus, all of a sudden you’ve lost all that spatial information. It is a very difficult chicken-and-egg problem as to what came first. Was it the wrapping of the nucleus that caused the actin and tubulin cytoskeletons to expand their capacities, or was it the explosion of the capacity of the cytoskeleton that wrapped up the nucleus in membrane? I like to imagine that at some point the nucleus got sequestered away somehow by some sort of prototypical membrane, maybe like what we see now in *Gemmata*, and then the poor little cytoskeletal elements were left out there in the cytoplasm on their own. They had no way of knowing where they were or of measuring space or position. So they had to figure out how to do it by themselves, without the chromosome there to help. Our eukaryotic cytoskeletons figured out how to do this by setting up large-scale arrays that can be oriented by virtue of having nucleators and molecular motor proteins to make those type B structures that are so useful for spatial organization over vast distances of many tens of micrometers. I think that this is a very elegant solution.

The other benefit that the eukaryotes may have gotten from this strategic decision is extra morphological evolvability. In one of your other interviews, Marc Kirschner made some very interesting points about how certain kinds of preexisting conditions may make it relatively easy for some animal lineages to generate highly variable morphology [108]. I think the eukaryotic cytoskeleton may well be an example of this at the cellular level, an idea that Marc also certainly shares [109]. Once the lonely but inventive eukaryotic cytoskeletal proteins committed to the strategy of using a very small number of filament types to perform a large number of different functions, the addition of a new kind of organizational function to the underlying cytoskeletal framework may have been as simple as coming up with a few new modulators of cytoskeletal filament dynamics, or another kind of slightly modified motor protein. This diversification may have happened very quickly on an evolutionary scale. Sequence analysis of the myosin and kinesin motor families seems to suggest that the most recent common ancestor for all the currently living eukaryotes already had several different kinds of each motor [110,111]. Indeed this most recent common ancestor may even have been capable of both amoeboid crawling motion and flagellar swimming [112]. It may be that the bacteria just never had to face this particular problem because, again, almost universally they have kept their chromosome right there in the cytoplasmic compartment where they could use it for spatial information. So typically, when a particular bacterium needs to make a filamentous structure for a novel purpose, such as orienting the magnetosomes in *Magnetospirillum*[5], it duplicates the gene for a cytoskeletal filament and adapts it for that one new purpose. This works fine for the purpose at hand, but forgoes the opportunity for flexibility and truly large-scale cellular organization that are intrinsic features of both the eukaryotic actin and microtubule cytoskeletons.

## Does that take us back to what the original eukaryotic cell might have looked like?

We’re certainly never going to know what the original eukaryote looked like. One major reason we’re never going to know is that all existing eukaryotes are very similar in many ways that must have come much, much later than that original separation of the eukaryotic lineage from the bacterial and archaeal lineages, suggesting that our most recent eukaryotic common ancestor was already quite a bit different from the original eukaryote and probably much more morphologically complex. So many of the most deeply rooted eukaryotic branches are just gone from the earth now, and we’re never going to see them. Knowing eukaryotes, I would guess that the ones that figured out how to do phagocytosis first just ate everybody else.

At some point initially, the earliest eukaryote must have looked much like its contemporary bacterial and archaeal counterparts, but it had secrets inside it that enabled it to become different. I think the fact that you see that both the diversification of the important NTPase families and the elaboration of cytoskeletal functions seem to be universal among eukaryotes means that probably those things happened relatively quickly. So when the lineage branched off, and maybe somehow the DNA got trapped in a nucleus and/or somehow membranes started being messed around with, that then generated a positive feedback loop that pretty quickly in evolutionary time caused it to turn into something with internal membrane-enclosed organelles and a mitotic spindle, and everything else we associate with eukaryotes came downstream of that. So I suspect the original eukaryote was small. I suspect it was pretty simple-looking compared with *Stentor* or one of the really fabulous single-celled eukaryotes.

## Also possibly simpler than the most complicated bacterium?

Certainly simpler than the most complicated bacterium. But with potential.
